# Sex differences in compulsive alcohol drinking phenotypes: implications for decision-making and social behavior in a preclinical model

**DOI:** 10.1007/s00213-025-06895-8

**Published:** 2025-09-16

**Authors:** Manuela Olmedo-Córdoba, José Juan León, Álvaro López-Villegas, Elena Martín-González, Margarita Moreno-Montoya

**Affiliations:** 1 Department of Psychology, Clinical and Experimental Neuroscience Research Group CTS280 and CIBIS (Centro de Investigación para el Bienestar y la Inclusión Social) Research Center, University of Almería, Ctra. Sacramento, s/n, 04120 Almería, Spain; 2 Área de Psicobiología, School of Health Science, Universitat Jaume I, Castellón de la Plana, Spain

**Keywords:** Alcohol drinking, Compulsive behavior, Social behaviors, Decision-making, Sexual dimorphism, Schedule-induced polydipsia, Cognitive processes, Phenotypes, Compulsive alcohol drinking, Preclinical model

## Abstract

**Rationale:**

Compulsivity is increasingly recognized as a transdiagnostic trait that amplifies vulnerability to alcohol use disorders. However, its specific role in shaping social behavior and decision-making remains underexplored.

**Objective:**

This study aimed to identify a vulnerable phenotype characterized by compulsive alcohol drinking and evaluate its behavioral alterations within the social behavior and cognitive processes domains of the Research Domain Criteria (RDoC), considering sex as a modulatory factor.

**Methods:**

Male and female Wistar rats were exposed to Schedule-Induced Polydipsia (SIP), first with water and then with alcohol. Distinct groups were formed based on intake patterns following a cluster-based analysis. We then assessed social subordination with the social dominance tube test (SDTT), sociability and social novelty with the three-chambered Crawley’s test (3CT), and decision-making with the rodent Gambling Task (rGT).

**Results:**

We identified four distinct behavioral profiles: Low Compulsive, Compulsive Alcohol, Compulsive Water, and High Compulsive. This segmentation revealed sex-specific distributions: males were overrepresented in high alcohol consumption clusters, while females were more prevalent in low-consumption profiles, indicating sex-related susceptibility. The High Compulsive phenotype diverged from the Compulsive Alcohol group, showing lower hierarchical status and a less risky decision-making strategy, whereas no significant differences were found in overall social interaction between groups. However, general alcohol consumption diminished general sociability and abolished sex differences, suggesting a disruption of innate social motivation.

**Conclusions:**

These findings support that the combination of compulsivity and alcohol intake increases behavioral vulnerability, specifically in domains of social competence and decision-making.

**Supplementary Information:**

The online version contains supplementary material available at 10.1007/s00213-025-06895-8.

## Introduction

Alcohol is the most widely consumed addictive substance globally, and its prolonged use can lead to alcohol use disorders (AUD), posing a major public health concern (Peacock et al. 2018). Epidemiological studies confirm that men are more likely to develop AUD and engage in chronic, compulsive patterns of alcohol consumption, whereas women tend to drink less; however, female alcohol use has significantly increased in recent decades, narrowing the gender gap (Bryazka et al. 2022; Erol and Karpyak 2015; Grant et al. 2017; Radke et al. 2021). According to the World Health Organization (WHO, 2024), approximately 2.6 million deaths were attributed to alcohol in 2019, with a higher prevalence among men. Addiction involves a transition from controlled use to compulsive consumption, marked by loss of control and persistence of behavior despite negative consequences (Robbins et al. 2012; Barker and Taylor 2014). While traits such as impulsivity and sensation-seeking have been extensively studied as vulnerability factors for problematic alcohol use (Belin et al. 2016; Giuliano et al. 2018), the role of compulsivity as a transdiagnostic trait remains less clearly defined. Clinical evidence suggests that compulsive traits may increase the risk for AUD, given their high comorbidity with obsessive compulsive disorder (OCD) (Osland et al. 2018; Virtanen et al. 2020).

Alcohol consumption has been associated with disruptions in social domains, including both social subordination and impaired social interaction. Social subordination, defined as occupying a lower hierarchical position within a group (Michopoulos et al. 2012), has been consistently linked to increased alcohol intake, largely mediated by chronic stress(Sapolsky 2005; Czoty et al. 2009; Bahi 2013). In humans, experiences of exclusion or discrimination correlate with higher alcohol consumption (Richman et al. 2002; Williams and Mohammed 2009), a pattern also observed in subordinate primates (McKenzie-Quirk and Miczek 2008; Helms et al. 2012b; Galbo et al. 2022). Alcohol also impairs social competence, defined as the ability to effectively apply social skills across different contexts (Kautz-Turnbull et al. 2023; Champagne et al. 2023). Animal studies indicate that this effect varies with age, facilitating sociability during adolescence but reducing it in adulthood(Varlinskaya and Spear 2007), and with sex, with males generally more susceptible to social deficits, although similar outcomes have been reported in females (Varlinskaya et al. 2014, 2017, 2020; Gamble and Diaz 2020; Towner et al. 2023, 2024; Bent et al. 2023).

In addition to these social disruptions, alcohol consumption has been associated with issues related to pathological gambling (Dowling et al. 2017; Allami et al. 2018). As observed in both patients with AUD and animal models, decision-making impairments, generally characterized as suboptimal, have been reported (Bechara et al. 2001; Fein et al. 2004; Kreek et al. 2005; Johnson et al. 2008; Kim et al. 2011; Voon et al. 2014). However, findings remain inconclusive, as studies in rodents and healthy humans have shown a range of outcomes, from impairment to no effect, or even improved performance under structured conditions (George et al. 2005; Perry and Carroll 2008; Mitchell et al. 2011; Peña-Oliver et al. 2014; Spoelder et al. 2017). Similarly, some clinical studies have reported comparable or even reduced risk-taking behavior in individuals with AUD (Ashenhurst et al. 2011; Claus and Hutchison 2012; Bø et al. 2016).

One of the most relevant preclinical models for studying compulsivity is Schedule-Induced Polydipsia (SIP), in which food-restricted animals exposed to an intermittent reinforcement schedule develop excessive and persistent adjunctive drinking behavior (Falk 1961; Moreno and Flores 2012; Moreno-Montoya et al. 2022; Martín-González et al. 2023a). This model has been validated using both water (Cardona et al. 2006; Moreno et al. 2010; Navarro et al. 2015; Merchán et al. 2019; Mora et al. 2020; Martín-González et al. 2022, 2023b) and alcohol (Mittleman et al. 2003, 2011; Escher and Mittleman 2006; Fouyssac et al. 2021; Galbo et al. 2022; Marti-Prats and Belin 2024), allowing animals to be classified into two phenotypes: High Drinker (HD), considered compulsive, and Low Drinker (LD), considered non-compulsive. HD rats exhibit behavioral alterations across multiple domains, including cognitive inflexibility (Navarro et al. 2017; Merchán et al. 2019; Prados-Pardo et al. 2019; Martín-González et al. 2023b), cognitive and motor impulsivity (Moreno et al. 2012; Merchán et al. 2019), risky decision-making (Martín-González et al. 2023b), and socioemotional disturbances (Martín-González et al. 2022; Prados-Pardo et al. 2023).

Preclinical and clinical evidence has linked compulsive spectrum disorders to alcohol addiction, with alcohol consumption associated with behavioral alterations across multiple domains. However, the specific role of compulsive alcohol intake in promoting maladaptive behaviors, particularly in the domains of cognitive control and social behavior, remains unclear. Additionally, little is currently known regarding potential sex differences in these effects. In this context, the general aim of this study was to identify a vulnerable phenotype characterized by a compulsive alcohol drinking pattern and systematically evaluate its behavioral alterations in cognitive control and the social domain, considering sex as a modulatory factor. To this end, we aimed to identify vulnerable phenotypes expressing one or both risk traits (compulsivity and/or alcohol consumption) on SIP model, and subsequently examine how these profiles impact risky decision-making, through the Rodent Gambling Task (rGT), and social functioning, using the Social Dominance Tube Test (SDTT) and the Three-Chambered Crawley’s Test (3CT). This approach enables the detection of compulsivity as a key vulnerability factor in the development of addiction and facilitates the identification of behavioral alterations with translational relevance.

## Materials and methods

### Animals

In this study, a total of 96 Wistar rats (48 males and 48 females) were used, sourced from Janvier Labs (Le Genest-Saint-Isle, France), and aged three months. The male rats weighed between 250 and 275 g, while the females weighed between 200 and 230 g. The animals were housed in groups of four per cage (50 × 35 × 20 cm) under controlled conditions, with a temperature of 23 ± 2 °C, humidity maintained at 50 ± 10%, and a 12-hour light/dark cycle (with the dark phase beginning at 07:00). Upon arrival, all animals were provided unlimited access to water and food, along with environmental enrichment, including wooden blocks. After an acclimatization period of 5 days, they were Handled daily for one week. Before starting the behavioral assessments, the animals underwent a gradual food restriction until they reached 85% of their free-feeding weight, which was then maintained throughout the study.

All procedures complied with ARRIVE guidelines and followed EU Directive 2010/63/EU and Spanish Royal Decree 53/2013 on the Care and Use of Laboratory Animals. The study followed the 3Rs principles (replacement, reduction, refinement).

### Experimental design

**Fig. 1:**
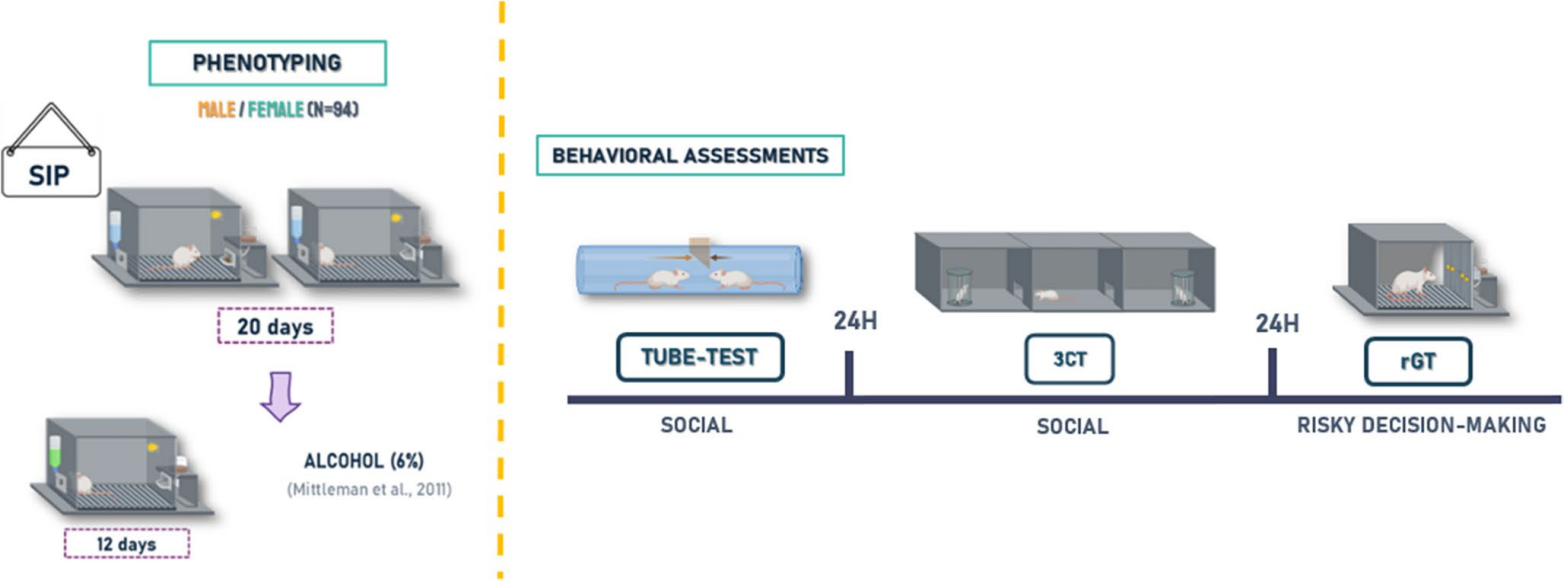
Experimental design. The experimental procedure is shown in a timetable. SIP, schedule-induced polydipsia; rGT, rodent gambling task; 3CT, three-chambered Crawley’s test. This figure was created with BioRender.com

*Phenotyping.* First, all animals were initially exposed to 20 sessions of SIP water acquisition. Once the compulsive behavior on SIP was established, water was replaced by alcohol for 12 sessions. Cluster analysis enabled the formation of four groups based on their consumption of water and alcohol: Low compulsive (low water and alcohol consumption), Compulsive alcohol (low water and high alcohol consumption), Compulsive water (high water and low alcohol consumption), and High compulsive (high water and high alcohol consumption). *Behavioral Assessment.* Subsequently, animals were assessed for sociability using the social dominance tube test (SDTT) and three-chambered Crawley’s test (3CT), and risky decision-making using the rodent gambling task (rGT). A timeline of the experimental events is presented in Fig. 1.

### SIP procedure

Before the SIP procedure, baseline water consumption was measured over two consecutive 60‑minute sessions during which rats had unlimited access to water and received 60 food pellets per session. SIPWater. Thereafter, animals underwent 20 SIP sessions in which 60 food pellets (Noyes 45‑mg dustless reward pellets; PHYMEP, France) were delivered on a fixed time schedule (FT‑60 s) during a 60‑minute session, while a 140‑160mL water bottle was available. SIPalcohol. Following the last SIP water session, the water was replaced with a 6% ethanol solution (Mittleman et al. 2003, 2011), and the SIP alcohol procedure was conducted for 12 sessions. The baseline assessment, allowing unlimited access to alcohol, was performed after completing the final SIP water session, aiming to prevent flavor aversion. The following measures were recorded for each rat: the total amount of water (milliliters) removed from the bottle, the total number of licks to the bottle, and the total number of entries to the food magazine (Mora et al. 2018).

### Behavioral assessment: social processes system

#### Social dominance tube test (SDTT)

Two classical tube tests were conducted using opaque PVC tubes featuring an opening at the top to facilitate the control of the animals. A victory was defined as when one animal succeeded in causing its opponent to place all four paws outside the tube, returning to its initial position. The procedure involved one day of habituation followed by three days of training. After completing the training, two conditions were evaluated: social dominance (against three unknown opponents of similar weight) and social hierarchy (with cage mates), totaling nine interactions per condition. The primary dependent variable was the percentage of victories per animal. Additionally, the transitivity of hierarchical relationships was assessed to determine the existence of a linear hierarchy. For further methodological details, see Supplementary Material and Perez-Fernandez et al. (2020); Martín-González et al. (2022).

#### Three-chambered crawley’s test (3CT)

The apparatus was divided into three chambers separated by transparent panels, allowing visual observation between compartments. The test included three phases: habituation, sociability, and reaction to social novelty. Following the criteria established by Bambini-Junior et al. (2014), rats that did not explore all three chambers during the sociability phase were not tested in the social novelty phase. Data were recorded for velocity (cm/s) and mobility (cm) in each zone, as well as accumulated time (s) and frequency of presence in each area of the apparatus. The indices were calculated as follows: (1) Social index (Time S1 – Time empty chamber)/(Time S1 + Time empty chamber); (2) Reaction to social novelty index (Time S2 – Time familiar chamber)/(Time S2 + Time familiar chamber) (Bambini-Junior et al. 2014; Perez-Fernandez et al. 2020; Morales-Navas et al. 2025). For more details on the procedure, please refer to the Supplementary Material.

### Behavioral assessment: cognitive control system

#### Rodent gambling task (rGT)

Animals were evaluated in six standard operant chambers. For 15 min, the rats were habituated to the testing environment, where reward pellets were available in the dispenser and response holes. Subsequently, they were trained to insert their noses into an illuminated hole within 10 s to obtain a reward. After meeting the criteria of ≥ 80% correct responses and ≤ 20% omissions, forced-choice training was conducted for 7 consecutive days in the rGT task to ensure balanced exposure to the reinforcement contingencies. The four options differed in reward magnitude and probabilities/durations of punishment: P1 delivered 1 pellet with a 90% probability of reward and a 10% probability of a 5-second time-out; P2 delivered 2 pellets with an 80% reward probability and a 20% probability of a 10-second time-out; P3 delivered 3 pellets with a 50% reward probability and a 50% probability of a 30-second time-out; and P4 delivered 4 pellets with a 40% reward probability and a 60% probability of a 40-second time-out (Zeeb et al. 2009).

During experimental sessions, each trial began with a nose-poke at the food dispenser, followed by a 5-second intertrial interval (ITI). Then, four holes were illuminated for 10 s. The task was designed so that P1 and P2 represented advantageous options (P2; the most optimal option), whereas P3 and P4 were disadvantageous choices (P4; the most disadvantageous). Correct responses were rewarded, while incorrect responses triggered punishment signaled by flashing lights. Premature responses during the ITI were punished with an additional 5-second delay, and perseverative responses during punishment or after reward were recorded but not penalized. Each new trial was initiated by a response at the dispenser. Sessions lasted 30 min and continued until stable choice patterns were observed across three consecutive sessions (for further details, see Zeeb et al. 2009; Tjernström et al. 2022a; Tjernström and Roman 2022b; Martín-González et al. 2023b). Further procedural details, including apparatus specifications, trial structure, reinforcement contingencies, and measured variables, are provided in the Supplementary Material.

### Statistical data analysis

#### Clustering approach

We employed hybrid hierarchical K-means clustering analyses to identify distinct drinking profiles based on the G/Kg of water and alcohol consumption in the SIP paradigm. For the cluster analysis, alcohol and water consumption data from the last 5 days were used. This was done to account for potential variations caused by the females’ menstrual cycle. This approach integrates the Ward’s linkage method on Euclidean distance with non-hierarchical K-means method to address the randomness in the initial selection of centroids (Hair et al. 2019). Our group has previously utilized this combination of clustering techniques to uncover new phenotypes in compulsive behavior (Merchán et al. 2019), decision-making (León et al. 2023), and attention-deficit hyperactivity behavioral profiles (Fernández-Martín et al. 2024). We posited the existence of four theoretical drinking profiles as reported by prior research (Fouyssac et al. 2021). This algorithm was applied to the standardized G/Kg of water and alcohol consumption in the entire sample. The optimal number of clusters was determined through dendrogram visualization and the majority rule of thirty clustering validation indices (Charrad et al. 2014). All analyses were performed using R software (R Core Team 2023).

#### Behavioral performance

Cluster-based and sex-level behavioral differences in the SDTT, 3CT, and rGT were tested using robust two-way ANOVAs with cluster membership and sex as between-subject factor. In phase 3 of 3CT, due to many exclusions, especially among males, analyses were limited to the factor Sex. ANOVAs were performed using the “welchADF” package (Villacorta 2017), using 10% trimmed means and winsordized variances as estimators for more robust hypothesis testing due to the characteristics of our sample. Critical and p-values are generated through bootstrap. Benjamini–Hochberg correction for multiple comparisons was applied when performing pairwise comparisons in order to control for Type-I error. Proportion tests were conducted to determine if the number of cases in each cluster relative to the total sample for sex differed from the expected values. Differences were understood as statistically significant when bootstrapped p-value < 0.05. All analyses were performed using R software (R Core Team 2023). All figures were made using GraphPad Prism 9.

## Results

### Phenotyping compulsive profiles

**Fig. 2:**
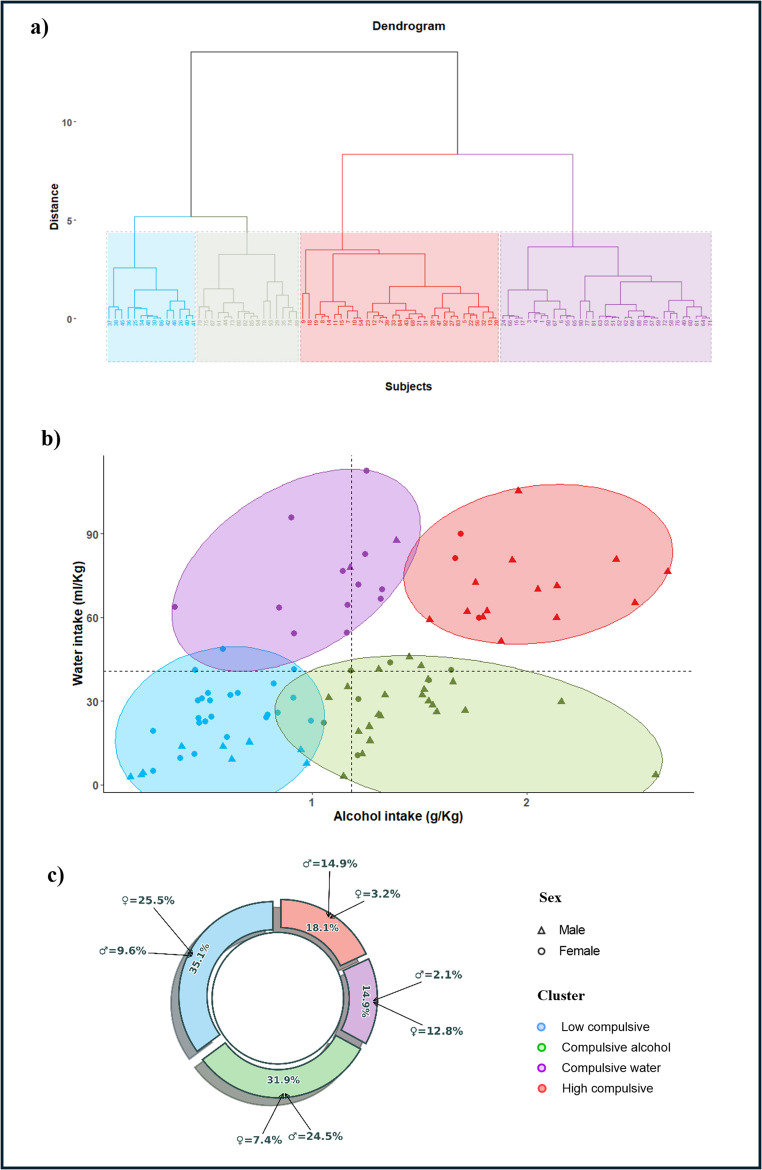
Distribution of subjects across four profiles based on water and alcohol consumption. Dendrogram from the hierarchical cluster analysis used to identify drinking profiles, illustrating the formation of clusters. (**b**) Final cluster solution showing the distribution of subjects by sex (males and females) within each identified profile. (**c**) Circular chart depicting the percentage of subjects in each cluster and the breakdown by sex within each cluster (♂ male and ♀ female)

The clustering approach identified an optimal solution consisting of four distinct drinking profiles (see Fig. 2).

The “Low compulsive” profile, which mainly included female rats, was noted for having both water and alcohol consumption levels beneath the average. The “Compulsive alcohol” profile, predominantly consisting of male rats, featured a combination of low water and high alcohol consumption. Additionally, the “High compulsive” group, also mainly male, demonstrated high consumption levels of both water and alcohol. Finally, the “Compulsive water” cluster, primarily comprising female rats, was recognized for its high water intake but low alcohol consumption. Subject distribution among the clustering measures is shown in Fig. 2a-b, and sex distribution within each cluster is presented in Fig. 2c and detailed in Table 1.

**Table 1:** Proportional distribution of subjects in each cluster according to sex

Cluster	Female ^a^	Male	Total	X² (*p*)
Low Compulsive	*n* = 24 (25.5%)	*n* = 9 (9.6%)	*n* = 33 (35.11%)	X² = 10.098, *p* = 0.001
Compulsive Alcohol	*n* = 7 (7.5%)	*n* = 23 (24.5%)	*n* = 30 (31.91%)	X² = 10.103, *p* = 0.001
Compulsive Water	*n* = 12 (12.8%)	*n* = 2 (2.1%)	*n* = 14 (14.89%)	X² = 7.259, *p* = 0.007
High Compulsive	*n* = 3 (3.2%)	*n* = 14 (14.9%)	*n* = 17 (18.09%)	X² = 6.674, *p* = 0.010
Total	*N* = 46	*N* = 48	*N* = 94	

^a^Two females were excluded due to the presence of outliers

### Compulsive phenotypes and their relationship with the social processes system

#### Social dominance tube test (SDTT)

Before conducting the SDTT analysis, transitivity (when animal A beats B, and B beats C, A must beat C) was assessed to validate the paradigm. In males, transitivity was 100% in dominance (100%) and 75% in hierarchy (75%), while in females, it reached 83% in both dominance (83%) and hierarchy (83%). Overall, high transitivity rates were observed across both sexes, with dominance (91%) and hierarchy (79%).

**Fig. 3:**
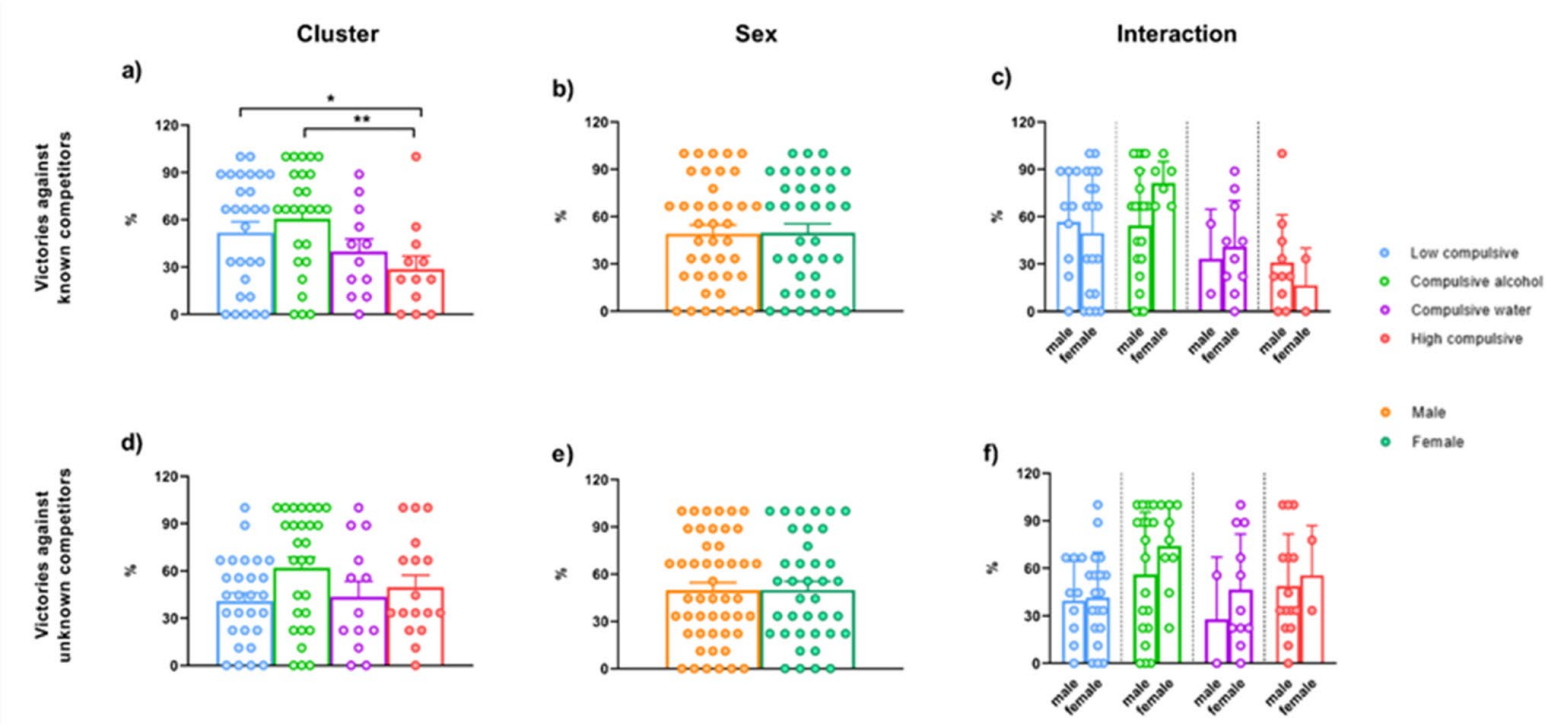
Social Dominance Tube Test. The percentage of victories against an unknown competitor (dominance) (**a**, **b**, **c**) or a known competitor (hierarchy) (**d**, **e**, **f**). [Cluster] * Significant differences between clusters

The percentage of victories against an unknown competitor (dominance) and against a known competitor (hierarchy) is presented in Fig. 3. Two-way ANOVA revealed a main effect of Cluster in Hierarchy (T_WJ_ (3, 16.45) = 9.951, *p* = 0.004). Pairwise comparisons showed that High compulsive rats seemed to be less dominant than Low compulsive rats (T_WJ_ (1, 39.71) = 10.541, *p* = 0.012, δ_R_ = −0.904) and Compulsive alcohol (T_WJ_ (1, 36.80) = 13.544, *p* = 0.004, δ_R_ = −1.072). However, no main effect of Cluster in the percentage of victories against an unknown competitor (T_WJ_ (3, 5.133) = 2.879, *p* = 0.196). Finally, regarding hierarchy, no main effect of Sex was found (T_WJ_ (1, 27.46) = 0.021, *p* = 0.891), nor Cluster x Sex interaction effect (T_WJ_ (1, 16.45) = 3.078, *p* = 0.056). Similarly, in dominance, no main effect of Sex (T_WJ_ (1, 4.348) = 1.742, *p* = 0.210) nor Cluster x Sex interaction effect (T_WJ_ (3, 5.133) = 0.505, *p* = 0.697).

#### Three-chambered Crawley’s test (3CT): motricity and velocity

**Fig. 4:**
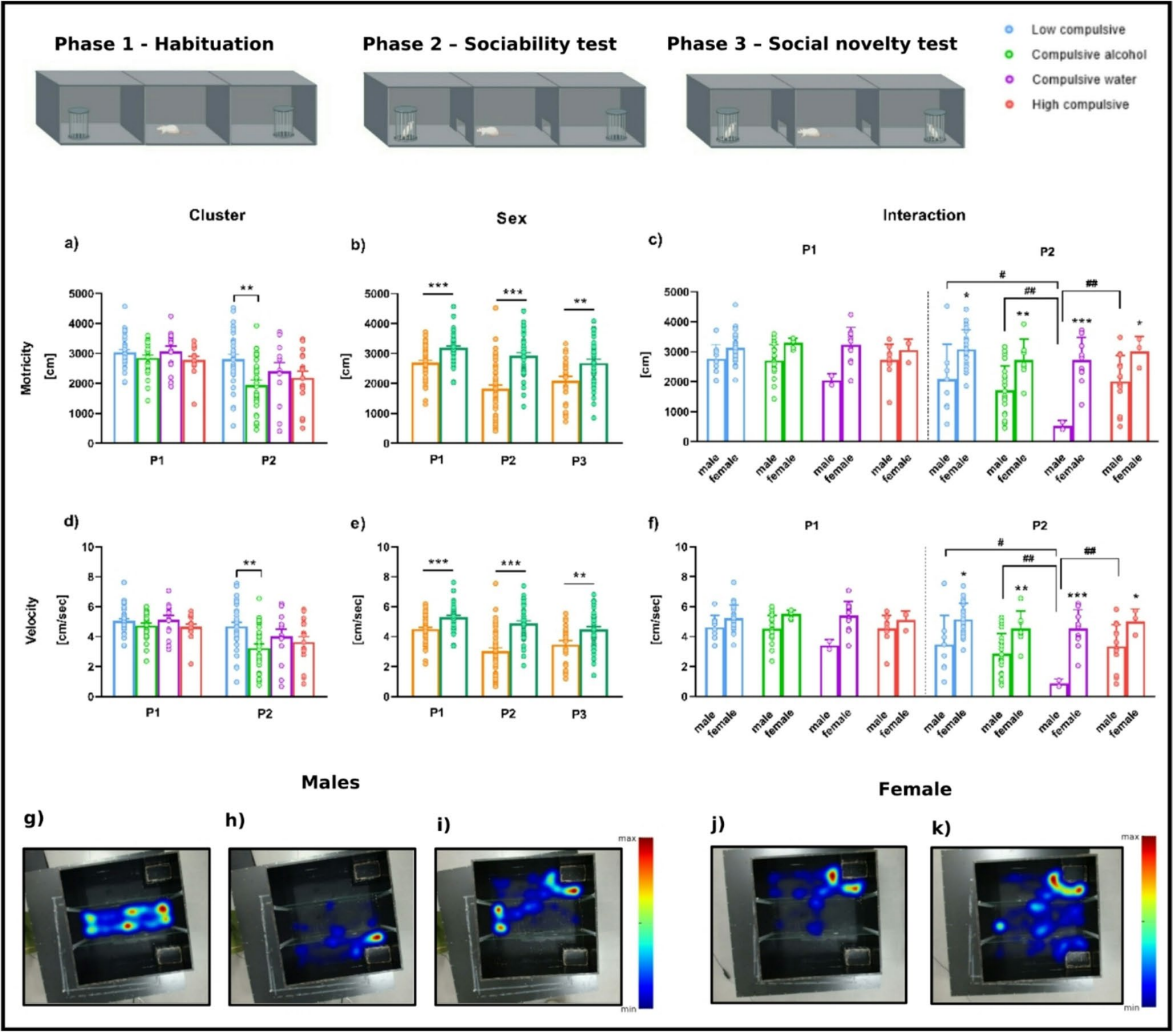
The 3-chambered test. Mean total distance traveled (motricity; **a**, **b**, **c**) and mean speed (velocity; **d**, **e**, **f**) in each phase. Heatmaps represent the density of time spent in each chamber during Phase 2 for males (g, h, i) and females (j, k). *Sample size*. Phase 1 (habituation) and Phase 2 (sociability test) [*n* = 94 (48 males and 46 females); Low Compulsive = 33 (9 males and 24 females), Compulsive alcohol = 30 (23 males and 7 females), Compulsive water = 14 (2 males and 12 females); High compulsive = 17 (14 males and 3 females)]. Phase 3 (social novelty test) [*n* = 69 (26 males and 43 females); Low Compulsive = 28 (5 males and 23 females), Compulsive alcohol = 18 (11 males and 7 females), Compulsive water = 11 (1 male and 10 females); High compulsive = 12 (9 males and 3 females)]. [Cluster] *Significant differences between different clusters. [Sex] *Significant differences between different sexes. [Interaction] *Significant differences within the same cluster. #Significant differences between males from different clusters

The total distance traveled (Motricity; [cm]) and velocity [cm/sec] during the three phases of the 3-chambered test are shown in Fig. 4. In Phase 1, a two-way ANOVA revealed a significant main effect of Sex on motricity (T _WJ_ (1, 38.415) = 38.415, *p* = 0.000) and velocity (T_WJ_ (1, 29.23) = 57.56, *p* = 0.000). As shown in Fig. 4b and e, during Phase 1, female rats exhibited a higher total distance traveled (T _WJ_ (1, 75.76) = 22.15, *p* = 0.000, δ_R_ = 0.967) and velocity compared to male rats (T_WJ_ (1, 75.74) = 22.09, *p* = 0.000, δ_R_ = 0.965).

In Phase 2, regarding motricity, a two-way ANOVA revealed a significant main effect of Cluster (T_WJ_ (3, 17.88) = 7.270, *p* = 0.007) and a Cluster × Sex interaction effect (T_WJ_ (3, 17.88) = 4.403, *p* = 0.018), driven by significant differences between sexes. Specifically, when isolating sex, Compulsive water male rats showed a lower motricity compared to Low compulsive (T_WJ_ (1, 8.966) = 14.837, *p* = 0.016, δ_R_ = −1.930), Compulsive alcohol (T_WJ_ (1, 9.134) = 25.601, *p* = 0.003, δ_R_ = −2.039), and High compulsive male rats (T_WJ_ (1, 10.100) = 29.723, *p* = 0.002, δ_R_ = −2.580) (Fig. 4c). Additionally, as shown in Fig. 4c, female rats exhibited a higher motricity compared to males across all groups: Low compulsive (T_WJ_ (1, 10.12) = 5.736, *p* = 0.037, δ_R_ = 0.941), Compulsive alcohol (T_WJ_ (1, 12.79) = 9.594, *p* = 0.009, δ_R_ = 1.145), Compulsive water (T_WJ_ (1, 8.238) = 79.83, *p* = 0.000, δ_R_ = 4.620), and High compulsive rats (T_WJ_ (1, 5.387) = 7.003, *p* = 0.042, δ_R_ = 1.290).

Regarding velocity, in Phase 2, the analyses indicated a significant main effect of Cluster (T_WJ_ (3, 17.80) = 7.208, *p* = 0.007) and a Cluster × Sex interaction effect (T_WJ_ (3, 17.80) = 4.401, *p* = 0.019). As shown in Fig. 4f, these differences are very similar to those observed for motricity. Specifically, Compulsive water male rats showed a lower velocity compared to Low compulsive (T_WJ_ (1, 8.967) = 14.781, *p* = 0.016, δ_R_ = −1.927), Compulsive alcohol (T_WJ_ (1, 9.069) = 25.600, *p* = 0.003, δ_R_ = −2.039), and High compulsive male rats (T_WJ_ (1, 10.073) = 29.724, *p* = 0.002, δ_R_ = −2.580). Also, female rats exhibited higher velocity compared to males across all groups: Low compulsive (T_WJ_ (1, 10.10) = 5.727, *p* = 0.038, δ_R_ = 0.941), Compulsive alcohol (T_WJ_ (1, 12.74) = 9.573, *p* = 0.008, δ_R_ = 1.145), Compulsive water (T_WJ_ (1, 8.241) = 79.56, *p* = 0.000, δ_R_ = 4.612), and High compulsive (T_WJ_ (1, 5.384) = 6.959, *p* = 0.043, δ_R_ = 1.287).

During Phase 2, in the male rat group (*N* = 48), only 26 rats exhibited activity in both chambers. The remaining 22 males did not meet the exploration criterion to proceed to Phase 3. Specifically, 9 rats spent time exclusively in the empty chamber without entering the stranger chamber (Fig. 4h), 10 entered the stranger chamber but not the empty one (Fig. 4i), and 3 remained in the central chamber without visiting either lateral compartment (Fig. 4g). In contrast, among the females, only 3 individuals failed to meet the criterion, and all of them explored the stranger chamber but not the empty one (Fig. 4j). Most females, however, showed activity in all chambers (Fig. 4k). Due to these results, Phase 3 is not discussed, as many of the clusters lack a sufficient number of subjects for analysis. Therefore, only sex differences were evaluated for this phase. Regarding velocity and motricity in Phase 3 (see Fig. 4b and e), the analyses revealed a significant main effect of Sex for both variables. Specifically, for velocity, an analysis statistic indicated a significant sex difference (T_WJ_ (1, 24.68) = 40.396, *p* = 0.000), with females showing higher velocity than males (T_WJ_ (1, 42.77) = 8.162, *p* = 0.006, δ_R_ = 0.710). Similarly, for motricity, there was also a significant main effect of Sex (T_WJ_ (1, 24.64) = 40.474, *p* = 0.001), again showing that females exhibited higher motor activity than males (T_WJ_ (1, 42.84) = 8.208, *p* = 0.006, δ_R_ = 0.712).

#### Three-chambered Crawley’s test (3CT): sociability

**Fig. 5:**
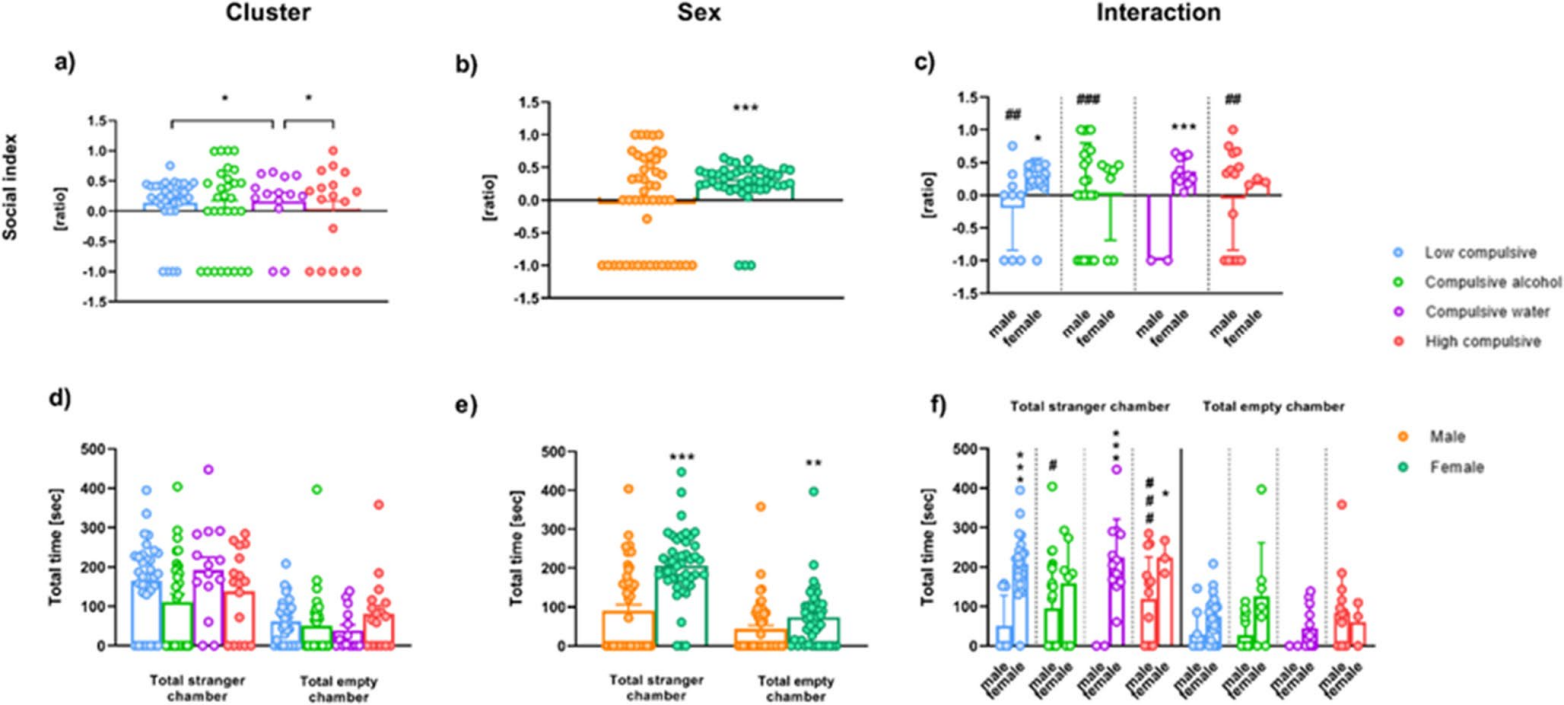
The 3-chambered test. Social index ratio (**a**, **b**, **c**) and mean total time spent in the stranger chamber and empty chamber (**d**, **e**, **f**) during Phase 2. *Sample size*. Phase 2 [*n* = 94 (48 males and 46 females); Low Compulsive = 33 (9 males and 24 females), Compulsive alcohol = 30 (23 males and 7 females), Compulsive water = 14 (2 males and 12 females); High compulsive = 17 (14 males and 3 females)]. [Sex] *Significant differences between different sexes. [Interaction] *Significant differences within the same cluster. #Significant differences between males from different clusters

The social index values are shown in Fig. 5. A two-way analysis revealed a significant main effect of Cluster (T_WJ_ (3, 19.30) = 7.162, *p* = 0.012), as well as a highly significant main effect of Sex (T_WJ_ (1, 32.25) = 20.377, *p* = 0.000). Additionally, the interaction between Cluster and Sex was also significant (T_WJ_ (3, 19.30) = 15.039, *p* = 0.005). Post hoc comparisons showed that, in males (Fig. 5c), the social index was significantly lower in Compulsive water group compared to Low compulsive (T_WJ_ (1, 8.00) = 13.863, *p* = 0.023, δ_R_ = −2.045), Compulsive alcohol (T_WJ_ (1, 18.00) = 33.440, *p* = 0.000, δ_R_ = −2.417), and High compulsive group (T_WJ_ (1, 11.00) = 15.372, *p* = 0.012, δ_R_ = −2.027). Comparisons between sexes within clusters revealed significant differences in Low compulsive group (T_WJ_ (1, 8.33) = 5.72, *p* = 0.043, δ_R_ = 1.112) and especially in Compulsive water cluster, where females showed a much higher social index than males (T_WJ_ (1, 9.00) = 479.6, *p* < 0.001, δ_R_ = 12.61).

The total time spent in the stranger chamber (placement of Stranger 1) and the empty chamber is shown in Fig. 5. This increased sociability is associated with differences in the total time spent in the stranger chamber. In this sense, a two-way ANOVA revealed a significant main effect of Sex (T_WJ_ (1, 29.23) = 57.56, *p* = 0.000) and Cluster × Sex interaction effect (T_WJ_ (3, 21.68) = 3.556, *p* = 0.039), driven by differences between sexes. As shown in Fig. 5f, female rats spent significantly more total time in the stranger chamber compared to male rats in the following groups: Low compulsive (T_WJ_ (1, 11.79) = 30.55, *p* = 0.000, δ_R_ = 2.043), Compulsive water (T_WJ_ (1, 9) = 117.8, *p* = 0.000, δ_R_ = 6.252), and High compulsive rats (T_WJ_ (1, 9.942) = 7.266, *p* = 0.023, δ_R_ = 1.237). Respect to male rats group, see Fig. 5f, Compulsive water males spent significantly less total time in the stranger compartment compared to Compulsive alcohol (T_WJ_ (1, 18.00) = 10.541, *p* = 0.023, δ_R_ = −1.356) and High compulsive male rats (T_WJ_ (1, 11.79) = 30.55, *p* = 0.000, δ_R_ = −2.043). Finally, concerning time spent in the empty chamber, a two-way ANOVA revealed a significant main effect of sex (T_WJ_ (1, 14.13) = 6.402, *p* = 0.028), with female rats spending more total time in the empty chamber compared to male rats (T_WJ_ (1, 73.48) = 7.574, *p* = 0.007, δ_R_ = 0.567) (Fig. 5e). The frequency of entries into each chamber is explained, along with the corresponding figure, in the Supplementary Material.

#### Three-chambered Crawley’s test (3CT): reaction to social novelty

**Fig. 6:**
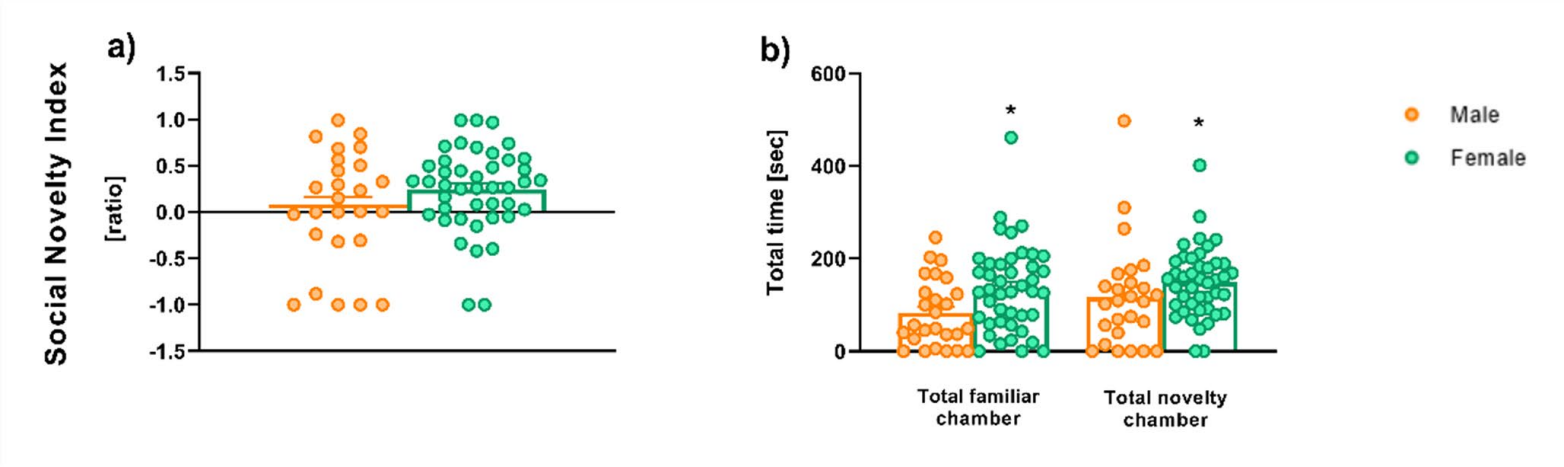
The 3-chambered test. Novelty index ratio (**a**) and mean total time in the familiar and novelty chamber (**b**) during Phase 3. *Sample size*. Phase 3 [*n* = 69 (26 males and 43 females); Low Compulsive = 28 (5 males and 23 females), Compulsive alcohol = 18 (11 males and 7 females), Compulsive water = 11 (1 male and 10 females); High compulsive = 12 (9 males and 3 females)]. *Significant differences between sexes

Figure 6 presents the social novelty index and the total time spent in the familiar chamber and the novelty chamber. The analysis revealed no significant effect of Sex on either the novelty index (T_WJ_ (1, 29.91) = 1.971, *p* = 0.176).

The time spent in the familiar and novelty chambers is shown in Fig. 6. As illustrated in Fig. 6b, a Welch’s *t*-test analysis revealed a significant main effect of Sex for the familiar chamber (T_WJ_ (1, 48.9) = 6.197, *p* = 0.012). Specifically, female rats spent significantly more time than males in this chamber (T_WJ_ (1, 48.9) = 6.197, *p* = 0.016, δ_R_ = 0.607). A similar pattern was observed in the novelty chamber, also shown in Fig. 6b, where the statistical analysis revealed a significant main effect of Sex (T_WJ_ (1, 32.99) = 4.179, *p* = 0.047), indicating that females spent more time in the novelty chamber compared to males (T_WJ (_1, 32.99) = 4.179, *p* = 0.049, δ_R_ = 0.535). No sex differences were observed in the frequency of entries into either the familiar or the novelty chamber. The corresponding figure is available in the Supplementary Material.

### Compulsive phenotypes and their relationship with the cognitive control system

#### Rodent gambling task (rGT)

**Fig. 7:**
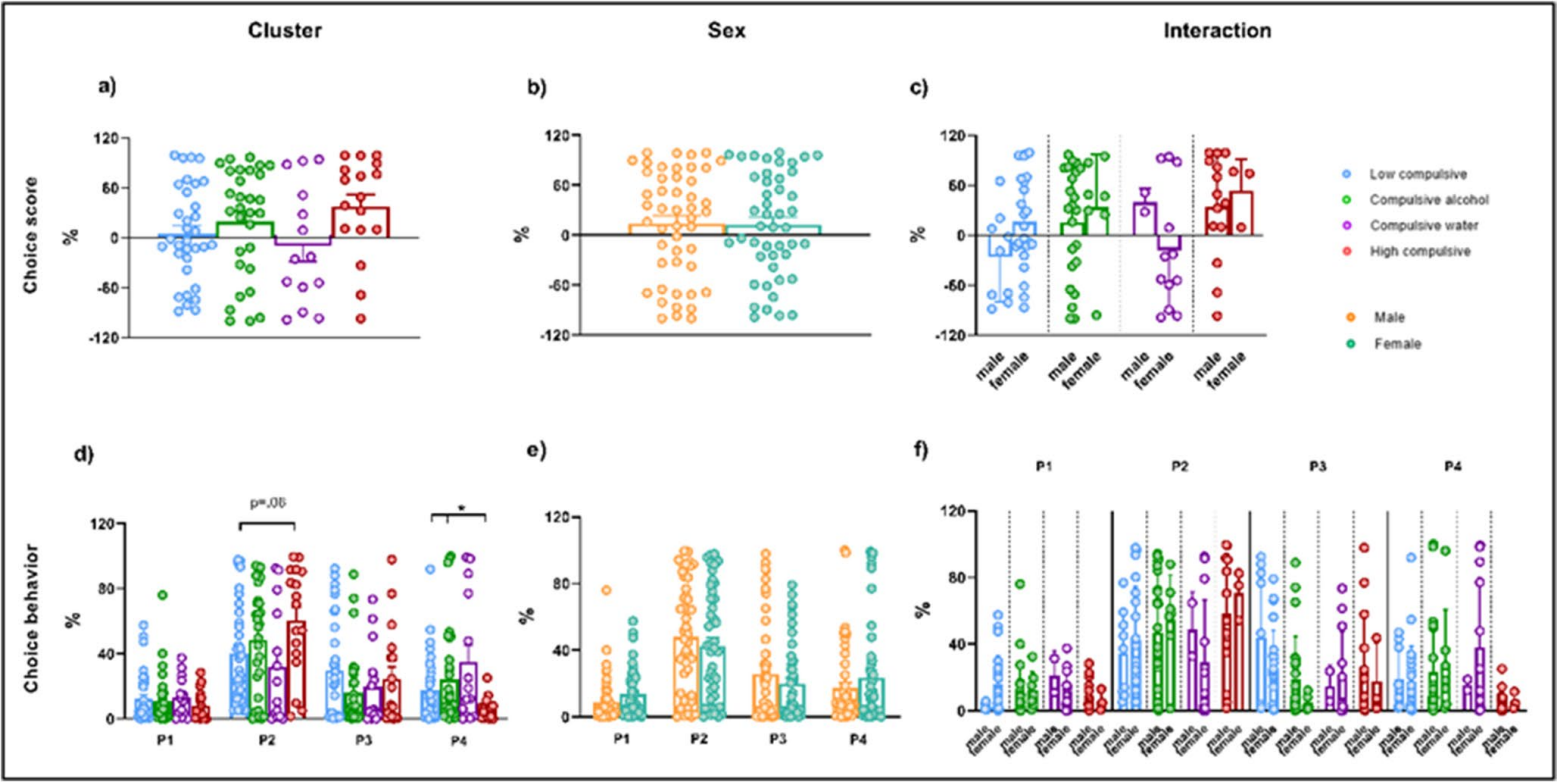
Rodent Gambling Task. The average percentage of the choice score (**a**, **b**, **c**) and the choice behavior (**d**, **e**, **f**), calculated from the mean of the last three experimental sessions. P1–P2 are advantageous options (P2 - the most optimal choice), whereas P3–P4 are disadvantageous options (P4 - the most disadvantageous). [Cluster]*Significant differences between clusters in each choice option

All dependent variables in the rGT were evaluated during the last three sessions to ensure the stability of the elections. As shown in Fig. 7, the average percentage of the choice score and the choice behavior at each specific probability were evaluated as dependent variables to explore the behavioral performance in the rGT. Regarding choice behavior, two-way ANOVA revealed a main effect of Cluster in the number of choices of P4 (T_WJ_ (3, 17.91) = 5.716, *p* = 0.036). Pairwise comparison showed that High compulsive rats showed less percentage of choices for P4 compared to Low compulsive (T_WJ_ (1, 34.04) = 7.324, *p* = 0.06, δ_R_ = −2.477) and Alcohol compulsive rats (T_WJ_ (1, 26.86) = 7.076, *p* = 0.06, δ_R_ = −0.841), although did not reach the significance threshold (Fig. 7d). Regarding the choice of P2, the analysis shows a trend in the main effect of Cluster (T_WJ_ (3, 12.19) = 3.312, *p* = 0.06). As shown in Fig. 7d, this trend indicates that High compulsive rats made a higher percentage of choices for P2 compared to Low compulsive rats (T_WJ_ (1, 22.518) = 14.062, *p* = 0.030, δ_R_ = 1.732).

**Fig. 8:**
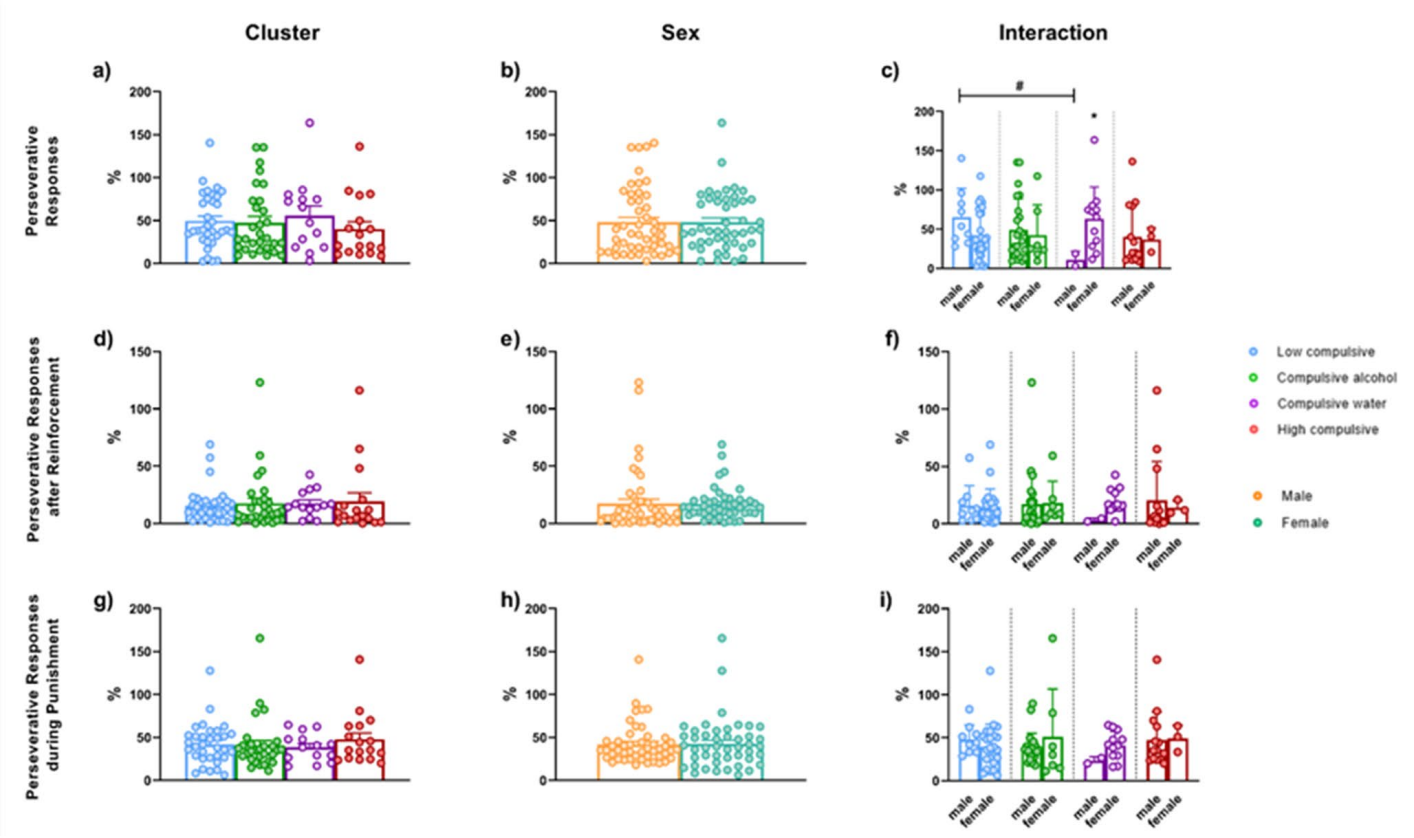
Rodent Gambling Task. The average percentage of the total perseverative responses (**a**, **b**, **c**), perseverative during the punishment (**d**, **e**, **f**), and perseverative after receiving the reinforcement (**g**, **h**, **i**), calculated from the mean of the last three experimental sessions. [Interaction] *Significant differences within the same cluster. #Significant differences between males from different clusters

Figure 8 displays the average percentage of total perseverative responses during the punishment and after receiving the reinforcement. Regarding total perseverative responses, a significant Cluster x Sex interaction effect emerged (T_WJ_ (3, 18.13) = 4.878, *p* = 0.014). When isolating Sex, as shown in Fig. 8c, Low compulsive male rats had more perseverative responses than Compulsive water male rats (T_WJ_ (1, 9) = 14.062, *p* = 0.046, δ_R_ = 1.879). When isolating Cluster, Compulsive Water female rats made more perseverative choices than Compulsive Water male rats (T_WJ_ (1, 4.547) = 15.72, *p* = 0.013, δ_R_ = 2.115).

Table 1, available in the Supplementary Material, presents the non-significant statistical analyses, as well as the effects of Cluster, Sex, and the Cluster x Sex interaction on the different variables used to explore behavior in the rGT.

## Discussion

The present study aimed to investigate whether compulsivity constitutes a behavioral trait associated with increased vulnerability to alcohol drinking, and how this vulnerability may manifest across social and cognitive domains, particularly in decision-making. To this end, we identified distinct behavioral profiles (Low Compulsive, Compulsive Alcohol, Compulsive Water, and High Compulsive) related to compulsivity and alcohol intake using a cluster-based approach. Our findings revealed a sex-dependent distribution: male rats were predominantly represented in the high alcohol consumption clusters, while females were more prevalent in the low-consumption profiles, suggesting sex-related differences in susceptibility to these phenotypes. Regarding behavioral differences across phenotypes, the data indicate that the High compulsive profile diverges from the Compulsive alcohol group, showing more significant negative impacts on both social competence and cognitive risk-related processing. This phenotype was marked by lower hierarchical status and a less risky decision-making strategy, whereas no significant differences were found in overall social interaction between groups. Taken together, these results support the notion that the combination of compulsivity and alcohol consumption increases behavioral vulnerability, impairing key domains such as social competence and decision-making, and positioning compulsivity as a transdiagnostic marker of behavioral risk.

### Phenotyping compulsive profiles

Our findings suggest that the differences between animals are not due to differential exposure but rather to a preexisting individual vulnerability trait. In the case of stress as an influencing factor, its effect on compulsivity (Yau and Potenza 2013; Ferreira et al. 2021) and alcohol consumption (Vengeliene et al. 2003; Siegmund et al. 2005; Sperling et al. 2010) is generally transient, disappearing once the stressor is removed. However, when stress is combined with an underlying vulnerability, it can consolidate addictive behavior into a stable trait (Nestadt et al. 2010; Koob and Volkow 2010). This concept is consistent with previous studies demonstrating that compulsive drinking induced by the SIP model, considered a stressful context, constitutes a stable and reproducible phenotype that persists over time even after re-exposure (Mora et al. 2020; Martín-González et al. 2022). This consistent pattern has also been observed with alcohol (Mittleman et al. 2003). Furthermore, high ethanol intake during the induction phase of the SIP model has been reported to predict subsequent chronic self-administration, indicating that initial drinking patterns under SIP are associated with a tendency to maintain elevated alcohol consumption when it is later available voluntarily (Grant et al. 2008).

Our cluster-based analytical approach enabled the identification of natural and stable profiles based on compulsivity and alcohol consumption in animals under the same conditions. Unlike previous classifications relying on predefined thresholds (e.g. Fouyssac et al. 2021; Marti-Prats and Belin 2024), this methodology offers a more accurate characterization of the role of compulsivity as a vulnerability factor for alcohol use. In relation to this individual differentiation, a seminal study by Wolffgramm (1995) already identified a subpopulation of male rats exhibiting compulsive alcohol consumption. Subsequent research has confirmed this phenotype, while also emphasizing that not all high-consuming animals develop compulsive alcohol-seeking behaviors (Vengeliene et al. 2009; Giuliano et al. 2015, 2018; Giuliano and Dalley 2020; Fouyssac et al. 2021). This evidence aligns with our group differentiation and supports the hypothesis that compulsivity, assessed during SIP water sessions prior to alcohol exposure, may act as a predisposing vulnerability factor contributing to the development of an addictive phenotype. However, the development of alcohol use disorders and addictive behaviors does not solely depend on basal compulsivity. Other motivational and biological factors also influence vulnerability to compulsive drinking (Gilpin et al. 2008; Ford 2014; Everitt and Robbins 2016)which may explain why some groups, such as Low Compulsive and Compulsive Water, do not drink alcohol.

Clinical evidence similarly supports a frequent comorbidity between OCD and AUD (Osland et al. 2018; Virtanen et al. 2020), suggesting that compulsive traits may contribute to vulnerability to problematic alcohol use (Gentil et al. 2009; Dwivedi and Kar 2017). Studies have shown that OCD often precedes the onset of AUD (Mancebo et al. 2009; Blom et al. 2011), and this relationship appears to be stronger among individuals with high anxiety sensitivity (Randazza et al. 2022). Alcohol may initially be used as a coping mechanism to mitigate intrusive thoughts, ultimately leading to a compulsive drinking pattern that exacerbates obsessive symptoms over time (Back and Brady 2008; Bakhshaie et al. 2021). Collectively, these findings support the notion that compulsivity precedes and facilitates the development of an addictive profile (Koob and Volkow 2010; Everitt and Robbins 2016).

Cluster-based group distribution revealed marked sex-related differences. Specifically, male rats were predominantly represented in clusters characterized by high alcohol consumption (Compulsive Alcohol) and by combined compulsivity and alcohol traits (High Compulsive). Conversely, female rats were minimally represented in alcohol-related clusters and were mostly concentrated in Low Compulsive and Compulsive Water groups. At the preclinical level, studies examining sex differences in alcohol consumption have yielded mixed results. Some reinforcement-based paradigms indicate higher compulsive self-administration in females under punishment (Siciliano et al. 2019; Domi et al. 2021, 2023), whereas other studies report greater alcohol motivation in males (Randall et al. 2017; Benvenuti et al. 2024). In mice, voluntary consumption paradigms such as Drink in the Dark generally report greater binge-like intake in females (Sneddon et al. 2019; Levine et al. 2021), although in Bauer et al. (2021) no sex differences were found. Overall, findings across various rat strains (Long-Evans, Wistar, Wistar Kyoto, Sprague-Dawley) report either similar alcohol consumption between sexes or inconsistent sex differences (Dhaher et al. 2012; Schramm-Sapyta et al. 2014; Priddy et al. 2017). Even in lines with high alcohol preference (e.g., HAD-1, HAD-2), results remain mixed, with some studies identifying sex differences (Mittal et al. 2019, 2020), while others do not(Dhaher et al. 2012; Haines et al. 2025).

Nonetheless, our results show a clear predominance of males in high-consumption profiles, aligning closely with clinical evidence. Historically, men have demonstrated a higher prevalence of problematic alcohol use (Bryazka et al. 2022; Wilsnack and Wilsnack 2013). Although alcohol consumption among women has increased in recent decades (Radke et al. 2021), men remain more likely to develop chronic and high-risk drinking patterns (Erol and Karpyak 2015). Furthermore, the male sex has been associated with greater prevalence and severity of AUD, as well as with comorbidity with other conditions such as OCD (Steketee 1997; Bystritsky et al. 2001). Altogether, our findings reinforce the translational validity of the model employed by reflecting patterns consistent with clinical observations.

### Social processes system

#### Social subordination

Our results show that the Compulsive Alcohol and Low Compulsive groups adopt a dominant social status, whereas the High Compulsive group displays a subordinate behavioral profile in familiar social contexts. This hierarchical distinction appears to be related to consumption patterns, as social subordination has been consistently linked to increased alcohol intake.

A classic study in primates already hinted at this relationship: following low doses of alcohol, aggressive behaviors increased, while higher doses led to reduced aggression (Winslow and Miczek 1985). Since then, this pattern has been replicated across various animal models. In rodents, both male and female subordinate rats and mice have been shown to consume more alcohol than their dominant counterparts (Ellison 1987; Blanchard et al. 1987; Wolffgramm and Heyne 1991; Kudryavtseva et al. 1991). Similarly, in primates, subordinate individuals have exhibited greater alcohol consumption compared to dominant ones, under both restricted and free-access conditions (Higley et al. 1996; McKenzie-Quirk and Miczek 2008; Helms et al. 2012a; Galbo et al. 2022; Galbo-Thomma et al. 2023).

Social subordination has been widely documented to induce chronic stress, which is often associated with increased alcohol consumption as a coping strategy. In primates, this phenomenon is reflected in heightened activation of the hypothalamic-pituitary-adrenal (HPA) axis and elevated cortisol levels in subordinate individuals (Sapolsky 2005; Czoty et al. 2009). Similarly, in rodents, prolonged subordination increases vulnerability to alcohol reinforcement due to its anxiolytic effects(Bahi 2013, 2017; Norman et al. 2015), a pattern also evident in social defeat paradigms (File et al. 1976; Spanagel et al. 1995; Macedo et al. 2018). In humans, social subordination, such as discrimination, exclusion, or unfair treatment, has been linked to increased alcohol consumption. This pattern is especially evident in vulnerable populations and may reflect a conserved cross-species mechanism (Mulia et al. 2008; Chae et al. 2008; Pascoe and Smart Richman 2009; Williams and Mohammed 2009).

In line with our findings, the High Compulsive group exhibited marked vulnerability to SIP-induced stress, as evidenced by a consistently high fluid intake regardless of its nature (Brett and Levine 1979; Dantzer et al. 1988; Mittleman et al. 1988). This sensitivity appears to extend to social behavior, with animals adopting a subordinate role in familiar settings. This aligns with literature linking compulsivity to emotional experiential avoidance and harm aversion (Bejerot et al. 1998; Den Ouden et al. 2020; Gillan et al. 2021). In contrast, the Compulsive Alcohol group displayed a clear preference for alcohol, maintaining low intake when water was concurrently available. This suggests that compulsive alcohol consumers exhibit a different social competence profile, potentially reflecting an avoidance oriented social strategy, compared to alcohol drinking animals without compulsive traits.

#### Social interaction

The results indicate that, in the 3CT, females exhibit a more pronounced sociability pattern compared to males, who tend to position themselves in the central or empty compartments, or even in the one containing the conspecific, but without engaging in interaction, thereby avoiding the contact zone. This behavior suggests that males display an inverted typical sociability pattern. Additionally, we observed that alcohol consumption attenuated these sex differences, as general sociability decreased and sex differences disappeared in the alcohol-consuming groups (“Compulsive Alcohol” and “High Compulsive”). This suggests that alcohol may interfere with social motivation by diminishing sensitivity to relevant social stimuli.

Sex differences in sociability have been extensively documented in animal models, although results vary depending on strain, task, and type of interaction evaluated. In rodents, under social operant conditioning tasks (where access to the stimulus is voluntarily chosen), females have demonstrated greater motivation to engage in social interactions, particularly in strains such as Wistar rats and C57BL/6J mice (Ramsey et al. 2022; Raymond et al. 2024). This effect may reflect increased sensitivity to social stimuli (Walker et al. 2009; Vidal et al. 2011). This pattern was replicated in the 3-chambered task by Socha et al. (2024), who observed greater sociability in female Wistar rats. In contrast, studies in Sprague-Dawley rats did not find sex differences (Venniro et al. 2018, 2019, 2020; Chow et al. 2022), reinforcing the influence of strain on social behavior (Varlinskaya 2008; Perkins et al. 2017; Kopachev et al. 2022).

Conversely, in paradigms involving forced physical contact, such as the social interaction test, males typically exhibit higher levels of social behavior. This pattern is particularly evident in strains like Lister Hooded, Sprague-Dawley, and Wistar rats (Johnston and File 1991; Stack et al. 2010; Graf et al. 2023). However, these differences can also be reversed in other strains, such as F344 rats, where females exhibit higher levels of social interaction (Perkins et al. 2017). Similarly, in social recognition tasks without direct contact, such as the social recognition test, females have demonstrated greater investigative exploration and retention of social memory (Markham and Juraska 2007), suggesting increased female sensitivity to subtle and novel social cues.

These differences are also reflected in humans, where it has been reported that women tend to process animated social interactions more rapidly (Pavlova et al. 2010), better recognize facial emotions (Fischer et al. 2018), and are more effective in interpreting subtle social cues, whereas men appear more oriented toward detecting threatening social signals (Kret and De Gelder 2012). Additionally, in populations with autism spectrum disorders, women often exhibit fewer difficulties in social interaction, better communication, and broader social networks (Mandy et al. 2012; Saure et al. 2023; Edwards et al. 2024; Finkel et al. 2024), supporting the existence of a profile of greater female social sensitivity shared across species.

Regarding alcohol consumption, our data show that it negatively modulates social interaction, reducing the sex differences observed in sociability. This reduction can be interpreted as an effect on social motivation, possibly decreasing inhibition or altering the response to novel social stimuli. Preclinical literature supports this finding: multiple studies have demonstrated that acute or repeated exposures to ethanol, especially during adolescence, induce behaviors similar to social anxiety and reduce social preference, with effects particularly consistent in males (Doremus-Fitzwater et al. 2009; Varlinskaya et al. 2014, 2017, 2020; Towner et al. 2023, 2024; Penta et al. 2025). It has also been observed that alcohol withdrawal in male rats significantly reduces social interaction (Brancato et al. 2021, 2023), even after three weeks of abstinence (Towner et al. 2023). However, similar effects have been reported in females. For example, Gamble and Diaz (2020) found that moderate alcohol exposure reduced both social preference and contact in adult females. Likewise, in rodent models, alcohol withdrawal has been observed to decrease social interaction in both sexes (Bent et al. 2022; Penta et al. 2025).

In the social novelty phase, the previously observed sex differences are mitigated. Female rats did not show a preference for the novel subject, suggesting a social selectivity oriented toward maintaining known bonds rather than exploring new ones. This pattern could reflect attachment behavior, consistent with human studies indicating higher attachment anxiety in women, while men exhibit higher levels of avoidance (Del Giudice 2011; Giudice 2019; Etchell et al. 2018; Han et al. 2024). This configuration suggests that women prioritize emotional closeness and are more sensitive to the absence of secure bonds (Collins and Read 1990). Thus, the lack of preference for novelty in females with high baseline sociability could represent a greater need for relational stability, opening avenues for future research on the interaction between attachment, sex, and social motivation in animal models.

### Cognitive control system

#### Decision-making

Our results indicate that the High Compulsive group made significantly fewer selections of the most disadvantageous option (P4) in the rGT and more frequently chose the advantageous option (P2), compared to the Low Compulsive and Compulsive Alcohol groups. This profile suggests a lower tendency toward risk-taking, likely driven by increased punishment avoidance. Notably, the Compulsive Alcohol group (with high alcohol consumption but without compulsivity toward water) did not differ from the control group in risky choices. This suggests that while alcohol consumption plays a role in risky decision-making, its impact becomes more pronounced when combined with generalized compulsivity, possibly due to heightened sensitivity to punishment that modulates decision-making behavior.

Although deficits in decision-making have been traditionally reported in individuals with AUD (Bechara et al. 2001; Fein et al. 2004; Johnson et al. 2008; Loeber et al. 2009; Gullo and Stieger 2011; Le Berre et al. 2014), experimental evidence remains inconsistent. Animal and human studies have shown mixed effects of alcohol on decision-making, ranging from impairment to improvement under specific conditions (George et al. 2005; Mitchell et al. 2011; DePoy et al. 2013; Schindler et al. 2014; Mejia-Toiber et al. 2014; Peña-Oliver et al. 2014; Spoelder et al. 2017). For example, (Spoelder et al. 2017) found that acute alcohol exposure increased preference for the optimal option in the rGT. Similarly, in humans, while some studies report clear deficits in tasks such as the Iowa Gambling Task (IGT) (Bechara et al. 2001; Fein et al. 2004; Na et al. 2019), others have found normal or even reduced risk-taking under structured conditions, such as the Balloon Analogue Risk Task and the IGT (Ashenhurst et al. 2011; Claus and Hutchison 2012; Bø et al. 2016).

Our findings may be interpreted within the framework of the alcohol myopia theory (Steele and Josephs 1990), which proposes that alcohol narrows cognitive processing, focusing attention on salient cues, thus promoting more conservative decisions. Supporting this, both clinical and preclinical research have demonstrated that the effect of alcohol on decision-making depends heavily on the clarity and visibility of negative outcomes. When these are explicit, individuals under the influence of alcohol tend to make more conservative choices, likely due to increased attentional bias toward punishment (McMurray et al. 2014; Carbia et al. 2017; Corbin and Cronce 2017; Wagner et al. 2018). In our study, punishment in the rGT served as a salient cue, as it was more severe in the disadvantageous options. This salience may have led High Compulsive animals to consistently avoid the most disadvantageous option (P4). Taken together, these findings highlight that the interaction between compulsivity, alcohol consumption, and sensitivity to negative outcomes may promote more conservative decision-making strategies in contexts where punishment is highly salient.

Some methodological considerations could help refine the interpretation of our findings. Periodic monitoring of the estrous cycle and quantification of sex hormones (estrogens, progesterone, testosterone) would clarify the extent to which hormonal fluctuations influence alcohol consumption and sociability. Testing multiple ethanol concentrations could reveal dose-dependent effects on compulsivity and social or cognitive behaviors, thus delineating the relationship between exposure level and deficit severity. Finally, ensuring balanced inclusion of males in the social novelty phase, either by adjusting the design to reduce exclusions in the social‐novelty phase or by adding a non‐SIP control group, would help disentangle ethanol’s effects from baseline sex differences.

## Conclusions

Our preclinical data establish compulsivity as a critical vulnerability factor for the development of alcohol intake. Cluster analysis on the SIP model identified a compulsive alcohol drinking phenotype (High Compulsive), predominantly in males, that exhibited social subordination on SDTT and with a conservative decision-making strategy in rGT, which might reflect greater sensitivity to punishment. In contrast, rats with high alcohol intake but without compulsive traits did not exhibit significant behavioral alterations in these dimensions. In social interaction, although females displayed higher sociability, these sex differences were attenuated by ethanol exposure, proposing that alcohol per se might disrupt innate social motivation regardless of compulsivity traits. Future research should interrogate the neurobiological substrates of these phenotypes, particularly alterations in gene expression, metabolomic signatures and HPA-axis dynamics. Such mechanistic insights will be essential for identifying biomarkers and designing targeted therapies for compulsive spectrum and AUD.

## Supplementary Information

Below is the link to the electronic supplementary material.

Supplementary Material 1

## Data Availability

All data reported in this paper will be shared by the lead contact upon request. Any additional information required to reanalyze the data reported in this paper is available from the lead contact upon request.
