# Supplementary material for: Sex differences in compulsive alcohol drinking phenotypes: implications for decision-making and social behavior in a preclinical model
Source: Psychopharmacology (Berl). 2025 Sep 16;243(5):1077–97. doi: 10.1007/s00213-025-06895-8 (PMC13242481; doi:10.1007/s00213-025-06895-8)
Supplement: Supplementary file 1 — Supplementary Material 1 [file 213_2025_6895_MOESM1_ESM.docx]

**PSYCHOPHARMACOLOGY**

**Sex Differences in Compulsive Alcohol drinking: Implications for Decision-Making and Social Behavior in a Preclinical Model**

Manuela Olmedo-Córdoba1, José Juan León1, Álvaro López-Villegas1, Elena Martín-González1, Margarita Moreno-Montoya1*.

**AFFILIATION**

**1** Department of Psychology, Clinical and Experimental Neuroscience Research Group CTS280 and CIBIS (Centro de Investigación para el Bienestar y la Inclusión Social) Research Center, University of Almería, Ctra. Sacramento, s/n, 04120, Almería, Spain

* Corresponding author: Margarita Moreno, Department of Psychology, University of Almería, Carretera de Sacramento s/n, 04120 Almería, Spain.

**e-mail**: mgmoreno@ual.es

**SUPPLEMENTARY INFORMATION**

**Materials and Methods**

**Social Dominance Tube Test (SDTT)**

The apparatus consisted of opaque PVC tubes with an opening at the top to allow for animal handling and monitoring during the task. The dimensions of the tubes differed by sex: 100 × 7 cm for males and 85 × 5.5 cm for females.

During the training phase, animals were gently guided through the tube using light pressure to encourage forward movement, and were rewarded upon exiting the tube in either direction. Each training session lasted approximately 5 minutes per animal. Following training, testing included two conditions—social dominance and social hierarchy—each composed of nine confrontations per animal. Between each confrontation, a 45-second interval was provided, and the apparatus was disinfected with 70% ethanol between animals to avoid olfactory cues or contamination.

**Three-chambered Crawley's test (3CT)**

The apparatus used for the three-chamber test was a rectangular box measuring 96 × 105 cm, subdivided into three equally sized chambers (96 × 35 cm): one central and two lateral compartments. These were separated by transparent glass panels that allowed visual observation between chambers. Each lateral chamber was further divided into a total zone and a contact zone, where the strangers are placed (Morales-Navas et al., 2024; Pérez-Fernández et al., 2020).

Animals were acclimated in the experimental room for 1 hour one day before the procedure. The tests consisted of three phases:

Phase 1: Habituation. The animal explored the central chamber freely for 10 minutes, allowing for the measurement of locomotor activity.

Phase 2: Sociability. Stranger 1 was introduced in a metal cage in one of the lateral chambers, allowing visual and olfactory contact for 10 minutes.

Phase 3: Reaction to Social Novelty. Stranger 2 was placed in the metal cage of the opposite lateral chamber, while Stranger 1 remained in its location, creating a social choice situation for another 10 minutes.

In each phase, the experimental animal was removed from the apparatus to implement necessary modifications. Both strangers were completely unknown to the experimental subject. After each trial, the apparatus was disinfected with 70% alcohol. The apparatus design and data recording were conducted using Ethovision 3.1 (Noldus).

**Rodent gambling task (rGT)**

The operant chambers used for the rGT were the same as those described in the SIP procedures, with the only modification being that five response holes were available, excluding the central one.

The forced-choice training phase lasted for 7 consecutive days and consisted of illuminating only one hole per trial to ensure that all animals received balanced exposure to the four different reinforcement contingencies associated with each choice option (P1–P4).

In experimental sessions, the contingencies were designed to ensure that P2 provided the optimal long-term payoff, while options with larger rewards were associated with more severe and frequent punishments, thus decreasing net gain over time. The spatial location of the four pellet choice options was counterbalanced across animals. In version A, the mapping of options P1–P4 to the holes followed one sequence, while in version B the order was altered to control for spatial bias.

The main measures included choice behavior, choice score, the proportion of perseverative responses during punishment, the proportion of perseverative responses after reward, and latency to respond and to collect the reward. For more details on the calculation of these variables, see Zeeb et al. (2009) and Martín-González et al. (2023).

**Results**

**Three-chambered Crawley's test (3CT)**

**Sociability**

The frequency of entries into the stranger chamber during Phase 2 revealed significant effects associated with both Cluster and Sex. A two-way analysis showed a significant main effect of Cluster (T_WJ_ (3, 22.30) = 5.460, p = 0.018), significant main effect of Sex (T_WJ_ (1, 38.66) = 32.061, p < 0.001), and a significant Cluster × Sex interaction (T_WJ_ (3, 22.30) = 7.122, p = 0.008). As shown in Figure 1c, female rats showed a higher frequency of entries into the stranger compartment compared to males in several clusters: Low compulsive (T_WJ_ (1, 9.36) = 7.478, p = 0.022, δ_R_ = 1.124), High compulsive (T_WJ_ (1, 11.19) = 5.781, p = 0.035, δ_R_ = 1.096), and Compulsive water group (T_WJ_ (1, 9.25) = 246.2, p = 0.000, δ_R_ = 8.591). Among male rats, post hoc comparisons revealed that Compulsive water group made significantly lower entries into the stranger chamber compared to those in the Compulsive alcohol (T_WJ_ (1, 18.08) = 16.206, p = 0.005, δ_R_ = 1.655) and the High compulsive group (T_WJ_ (1, 11.04) = 12.955, p = 0.021, δ_R_ = 1.828).

In contrast, for the frequency of entries into the empty chamber, no significant effect of Cluster was observed (T_WJ_ (3, 11.60) = 2.138, p = 0.201), nor was there a significant Cluster × Sex interaction (T_WJ_ (3, 11.60) = 1.184, p = 0.380). However, a significant main effect of Sex was found (T_WJ_ (1, 11.32) = 7.500, p = 0.015), indicating that female rats made more entries into the empty chamber than male rats (T_WJ_ (1, 11.32) = 7.500, p = 0.019, δ_R_ = 2.71).


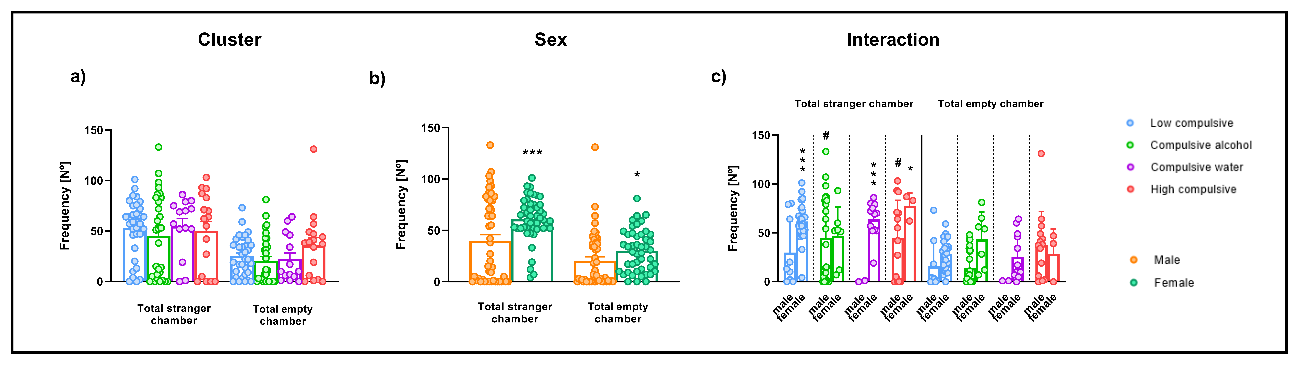


**Supplementary Fig.1** Frequency in the stranger and empty chamber of the 3-chambered test.  Phase 1 and Phase 2 [n = 94 (48 males and 46 females); Low Compulsive = 33 ( 9 males and 24 females), Compulsive alcohol = 30 (23 males and 7 females), Compulsive water = 14 (2 males and 12 females); High compulsive = 17 (14 males and 3 females)]. [Sex] *Significant differences between different sex. [Interaction] *Significant differences within the same cluster. #Significant differences between males from different clusters

**Reaction to social novelty**

The analysis of the frequency of entries into familiar and novelty chambers during Phase 3 did not reveal significant differences between sexes (see Figure 2). For the familiar chamber, the effect of Sex was not significant (T_WJ_ (1, 29.65) = 0.315, *p* = 0.553), and similarly, no sex differences were found in the frequency of entries into the novelty chamber (T_WJ_ (1, 31.69) = 0.730, *p* = 0.416).


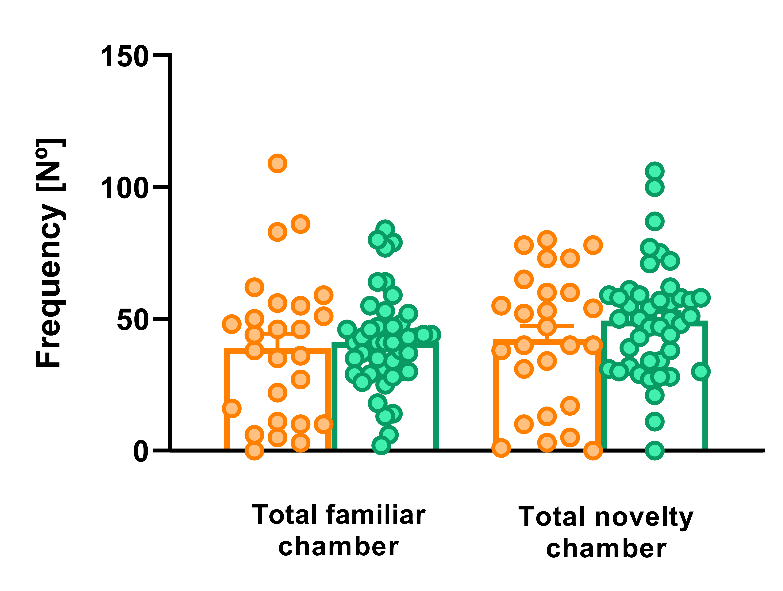


**Supplementary Fig. 2** Frequency in the familiar and novelty chamber of the 3-chambered test. Phase 3 [n = 69 (26 males and 43 females); Low Compulsive = 28 (5 males and 23 females), Compulsive alcohol = 18 (11 males and 7 females), Compulsive water = 11 (1 male and 10 females); High compulsive = 12 (9 males and 3 females)]

**Rodent gambling task (rGT)**

| **Variable rGT** | **Effect Principal** | **F-Test** | **P-Value** |
| --- | --- | --- | --- |
| % Choice score | Cluster | 2.755 | 0.09 |
|  | Sex | 0.041 | 0.866 |
|  | Cluster x Sex | 2.673 | 0.086 |
| %P1 | Cluster | 0.734 | 0.614 |
|  | Sex | 0.123 | 0.727 |
|  | Cluster x Sex | 1.769 | 0.407 |
| %P2 | Cluster | ------ | ------- |
|  | Sex | 0.001 | 0.977 |
|  | Cluster x Sex | 0.608 | 0.640 |
| %P3 | Cluster | 2.717 | 0.142 |
|  | Sex | 1.147 | 0.173 |
|  | Cluster x Sex | 0.520 | 0.675 |
| %P4 | Cluster | ------ | ------- |
|  | Sex | 1.517 | 0.233 |
|  | Cluster x Sex | 0.907 | 0.454 |
| Perseverative responses | Cluster | 0.829 | 0.337 |
|  | Sex | 1.706 | 0.210 |
|  | Cluster x Sex | ------ | ------ |
| Perseverative responses during Punishment | Cluster | 2.028 | 0.207 |
|  | Sex | 1.474 | 0.256 |
|  | Cluster x Sex | 2.772 | 0.161 |
| Perseverative responses after reinforcement | Cluster | 0.482 | 0.716 |
|  | Sex | 1.876 | 0.189 |
|  | Cluster x Sex | 3.363 | 0.10 |

**Supplementary Table 1.** Statistical analysis on the Rodent Gambling Task (rGT). The effects of Cluster, Sex, and the Cluster x Sex interaction on the different variables used to explore behavior on rGT. Data are expressed with the F-test and P-values.  **--**Significant data are presented in the results
